# An Intelligent Vision Based Sensing Approach for Spraying Droplets Deposition Detection

**DOI:** 10.3390/s19040933

**Published:** 2019-02-22

**Authors:** Linhui Wang, Xuejun Yue, Yongxin Liu, Jian Wang, Huihui Wang

**Affiliations:** 1 College of Electronic Engineering, South China Agricultural University, Guangzhou 510642, China; Jetwlh@stu.scau.edu.cn; 2 The Department of Electrical, Computer, Software, and Systems Engineering, Embry-Riddle Aeronautical University, Daytona Beach, FL 32114, USA; ctliuyongxin@mail.scut.edu.cn (Y.L.); wangj14@my.erau.edu (J.W.); 3 The Department of Engineering at Jacksonville University, Jacksonville, FL 32211, USA; hwang1@ju.edu

**Keywords:** droplets, intelligent node, vision sensor, adaptability, Unmanned Aerial Vehicles

## Abstract

The rapid development of vision sensor based on artificial intelligence (AI) is reforming industries and making our world smarter. Among these trends, it is of great significance to adapt AI technologies into the intelligent agricultural management. In smart agricultural aviation spraying, the droplets’ distribution and deposition are important indexes for estimating effectiveness in plant protection process. However, conventional approaches are problematic, they lack adaptivity to environmental changes, and consumes non-reusable test materials. One example is that the machine vision algorithms they employ can’t guarantee that the division of adhesive droplets thereby disabling the accurate measurement of critical parameters. To alleviate these problems, we put forward an intelligent visual droplet detection node which can adapt to the environment illumination change. Then, we propose a modified marker controllable watershed segmentation algorithm to segment those adhesive droplets, and calculate their characteristic parameters on the basis of the segmentation results, including number, coverage, coverage density, etc. Finally, we use the intelligent node to detect droplets, and then expound the situation that the droplet region is effectively segmented and marked. The intelligent node has better adaptability and robustness even under the condition of illumination changing. The large-scale distributed detection result indicates that our approach has good consistency with the non-recyclable water-sensitive paper approach. Our approach provides an intelligent and environmental friendly way of tests for spraying techniques, especially for plant protection with Unmanned Aerial Vehicles.

## 1. Introduction

It is known that there is a momentum in the new technological revolution and the new industrial revolution. We believe that the new era of AI, which is characterized by ubiquitous networks, data-drivenness, shared services, cross-border integration, automatic intelligence, and mass innovation, is coming soon. Among these characteristics, machine vision is a branch of the rapid development of AI, which is based on vision sensor systems. As computer science develops, vision sensor based on AI is gradually changing many aspects of human life and making our world smarter and smarter. Among these trends, improving the intelligent management of agriculture is of great significance.

Spraying pesticide is one of the most significant approaches of plant protection during the process of preventing diseases and pests. In order to avoid the drawbacks of the traditional approaches such as high labor intensity and low efficiency, the technologies of agricultural aviation represented by the Unmanned Aerial Vehicles (UAVs) have been gradually developed [1]. For a long time, the researchers found that, due to the influence of nozzle performance, meteorological conditions, characteristics of crop leaf surface and so on, the pesticide liquid, which is sprayed out from the nozzle in UAVs spraying work, has a series of phenomena, such as droplets shifting, droplets bounce/crushing, droplets gathering and droplets deposition. And the pesticide spraying quality is determined by the deposition of pesticide liquid on the target object. Therefore, the precise measurement of droplets parameters and the distribution of droplets mastering are of great significance to better controlling the spraying process [2].

Although many researches have been conducted on how to accurately determine the droplets parameters of UAVs spray and how to evaluate the deposition rules, essentially, they can be divided into quantitative analysis and qualitative analysis. Quantitative analysis refers to the direct measurement of the deposition parameters of pesticide liquid by means of measuring equipment, either manually or mechanically. In [3,4,5,6], different quantitative analysis methods have a higher detection accuracy, but there are also the following key defects: (1) complicated implementation process, and high risk of error caused by human factors; (2) Expensive and cumbersome equipment, low intelligent level, and a lack of scientific testing process; (3) Poor adaptability and harsh environmental requirements. It is mainly reflected in the fact that it is difficult to adapt to the changing environmental light source and the sampling carrier is easy to be contaminated by water, oil, and other substances; (4) Slow detection speed, not suitable for large-scale droplets detection. Qualitative analysis mainly refers to the research on the measurement method of droplets based on computer vision, and it can rapidly complete the measurement and analysis of droplets and acquire the quantitative index of droplets parameters, which has become an important research direction of spray technology [7]. The existing qualitative analysis methods mainly adopt paper materials such as water-sensitive paper and coated paper as the droplets test card to take the droplets, and then the edge extraction as well as open/closed operation or color distortion to realize droplets image segmentation, so as to acquire relevant deposition parameters of spraying droplets. But the cost of these methods is higher, and their material utilization ratio is low and timeliness is strong. The key point is that the existing droplets collection equipment is not intelligent enough to realize one-click fast collection and automatically adapt to the environment illumination.

To alleviate the problems caused by above-mentioned approaches. In this paper, we design a new intelligent node based on vision sensor for automatic and circular gathering of droplets deposition images combining with T-310 achromatic ink, which has water-transparent and dry-rejuvenating properties. Under the circumstance that illumination values are different, and the thickness of the ink remains unchanged, but the contrast of the droplets image changes, reinforcement learning algorithm is adopted to realize the adaptive adjustment of the contrast parameters of droplets. On the basis of morphology, the shape degree and area threshold are introduced as the basis for the determination of adhesion, and the modified marker controllable watershed segmentation (MMCWS) algorithm is proposed to segment the adhesive droplets. Based on this, statistics and calculation of the droplets’ quantity, coverage, coverage density and other parameters on the image of the achromatic oil-paper which is a transparent silicone oil paper with T-310 achromatic ink on single-faced are conducted. Finally, a practical application which is tested in a playground for gathering droplet image data is implemented, and then it is analyzed to verify the feasibility of our approaches.

Our works’ major contributions are highlighted as below:

1. We explored a novel intelligent droplets collection node, which is an automatic control and closed device with less environment interference. What’s most important is that our node can adapt to different lighting conditions and large-scale detection for aviation droplet, and avoid the human errors existing in common detection approaches.
2. Combining with T-310 achromatic ink which has water-transparent and dry-rejuvenating properties, we design T-310 achromatic oil paper based on silicone oil paper as a droplet deposition carrier, which can repeatedly collect droplets image, and make up for some disadvantages of the common droplets carriers with a low utilization rate, high cost, and easy contamination.
3. On the basis of the morphology, we introduce the shape degree and area threshold as the basis for the determination of droplets’ adhesion, and then modify the marker-controlled watershed segmentation algorithm to segment the adhesive droplets.

The rest of this paper is organized as follows. Section 2 reviews the related works. Section 3 introduces the intelligent detection node and mathematical algorithms. Models validation results and real-world experimental analyses are reported in Section 4, followed by a conclusion in Section 5.

## 2. Related Works

The parameters of UAVs droplets deposition are an important index which determines the mechanical properties of aerial plant protection. With the in-depth understanding of the impact of the droplets deposition parameters on their performance as well as the great attention paid to the problems caused by the droplets drifting, more and more attention is paid to the research on the measurement technology of droplets deposition parameters. Rapidly and accurately measuring droplets parameters and mastering the droplets deposition law are indispensable preconditions for the development, test, production, and use of plant protection machinery, and they are of great significance for better understanding and controlling the spraying process. Common quantitative analysis approaches such as high-speed photography [8], laser holography [9], laser imaging [10], and fluorescent tracer [11,12,13] could directly measure droplets size and distribution law, but the sampling data of these methods have a poor continuity, complicated handling mode, and high operation difficulty, so they aren’t applicable to the large-scale rapid detection of droplets in the field.

Computer vision technology is not only an essential part of artificial intelligence but also a challenging topic in the field of science. Today, the continuous development of computer vision technology is promoting the progress of visual inspection, which involves in image processing, pattern recognition, automatic control, cyber-physical systems [14], artificial intelligence and many other disciplines. Therefore, a large number of people think applying computer vision toanalyze aviation droplets image quickly is an inevitable trend. Hermosilla [15] used the adaptive threshold approach for the binarized water-sensitive paper image and measuring actual size of the droplets by CCD software, and then classified it into linear regression analysis and statistical processing. It was found that the average relative error of coverage was 5.15%. This approach reduced the operator’s subjective factors and the segmentation error. Qi [16] adopted water-sensitive paper to collect droplets, gather images of droplets through image acquisition system and perform the analysis, and the acquire the parameters such as droplets size distribution and coverage, which were compared with those of laser particle size analyzer with an error of less than 6%. Zhang [17] tested the effective width and uniformity of droplets deposition under the upwind conditions with water sensitive paper and self-developed image recognition system software. However, the process of the above scheme was separated. Namely, the droplets image was first collected outside, and then the image analysis was carried out indoors, which failed to achieve the integrated black box processing. At present, the research on integrated intelligent droplets detection equipment that collects, processes and outputs the droplets deposition parameters in real time have not yet been reported.

When vision equipment senses droplets information through the camera, the image quality is affected by many factors, such as the nature of droplets carrier material, the degree of refraction and reflection to light, the degree of light intensity, the hardware, etc., of which the impact of illumination change on the acquisition of droplets images by the camera is particularly apparent. The main impact is reflected in two aspects: (1) the poor visual effect of the images seriously affects the observation of droplets by the visual system; (2) changes in illumination can lead to the changes in some features of droplets image, thus affecting subsequent image segmentation, feature extraction, etc. [18]. Therefore, a good droplets detection vision system should have a strong robustness and adaptability dealing with changes in illumination. For this purpose, many methods have been tried, such as feature comparison method, the background subtraction method, and camera parameter self-calibration method. However, the methods as mentioned above only improved the adaptability of ambient light to some extent and under some circumstances. Reinforcement Learning is a bionic algorithm designed by scientists according to the inspiration of animals’ self-learning process, and it can enable an autonomous agent perceiving the environment to select the action which can achieve the optimal goal through the self-learning process similar to animals. Comparing with the Q learning, the previous state Q in the Sarsa learning is real-time rather than the maximum value of the cumulative Q when it is updated, so the Sarsa learning can avoid that it’s search behaviors always go straight to the direction with the largest Q. ignoring the optimal strategy hidden in other directions [19]. Based on this, under the circumstance that the same object remained color chromaticity range unchanged under different illumination values, but the degree of saturation and brightness range changed, literature [20] adopted Sarsa learning to achieve an adaptive adjustment of the color saturation and brightness parameter values of the target body, and it had a better adaptability and robustness to the acquisition of the target body in an environment of changing illumination. This research work provided a positive reference for the self-adaptability of the vision system under ambient light described in this paper.

The key of computer visual inspection of droplets is to acquire the parameters of droplets on droplets carrier image through image processing and other technologies, and the main steps include image preprocessing, image segmentation, pattern recognition, and parameter extraction. Among them, image segmentation is one of the critical steps, and the accuracy of segmentation is directly related to parameter extraction. Common image segmentation methods include clustering method, threshold segmentation, fuzzy set method, and watershed method. For example, Yang [21] took advantage of K-mean clustering algorithm in the L*a*b space to acquire the target area of droplets, and then adopted the compound method of open and close operation to filter the image after clustering for multiple times, and then completed the segmentation of the target. This method can better segment the droplets from the image, but it is easy to ignore the small droplets, and it involves in open and close operation, so the size of droplets measured by it has a deviation from the true value. If the object has a clear boundary, a simple threshold method can segment the object from the background very well. But if the droplets are adhesive, such methods have a limited effectiveness. Watershed method is an effective tool to solve such problems. The original watershed directly adopts the minimum value of the area or the final erosion point as the starting point, and it can segment the adhesive droplets, but over-segmentation may occur at the same time [22]. The marked controllable watershed segmentation method proposed by literature [23] adopts the pre-defined marker to replace the minimum value of the region or the final erosion point, and each marker represents an object, which can effectively solve the problem of over-segmentation, but this method cannot automatically identify the adhesive object. Therefore, this paper first made a physical judgment on droplets, and then adopted the marked controllable watershed method to segment the adhesive droplets.

In the process of detecting spraying performance and measuring the size of droplets with computer vision technology, the overlapping and adhesion of droplets often occurred in the image of droplets. For the determination of adhesive droplets, many methods have been explored, such as regional growth method [24] based on color similarity and meeting the “convexity” criteria, and the approximate concave point cutting method of binary image polygon [25], but they have some limitations in practical processing. Inspired by algorithm that the separation point processed overlapping red blood cells in literature [26], Qi [16] found the shape of normal single droplet was nearly a circle, but the shape of adhesive droplets became irregular, and the separation points usually appeared on both sides of the long axis, so the circular degree was introduced as a characteristic quantity to calculate the complexity of object shape, and then an improved adhesive droplets decision method was put forward, and it improved the separation accuracy. However, the algorithm only judged the adhesive droplets by the circularity. Although the droplets were segmented, the overlapping small areas were eliminated. Based on Qi’s approach, Wu [27] used the limit corrosion approach and iterative opening operation to realize the droplets counting, and then introduced the watershed of the iterative opening operation to segment the droplets. Finally, the area marking angle recognition algorithm was applied to shape the circle, and the results indicated that the proposed approach could effectively compensate for the loss of detection in small areas, and at the same time, the detection accuracy was 7.77% higher than that of Deposit Scan software (USDA-ARS Application Technology Research Unit, Wooster, Ohio, USA, DepositScan 1.0). Therefore, this thesis used a part of Wu’s adhesive droplets judgement approach for reference when solving the problem of droplets adhesion.

Common sample measurement approaches are defined as using droplets to collide with a carrier to leave a mark on their surface, and then the size of the droplets is measured by a microscope, but with a high work intensity and low precision [28], selecting a suitable droplets collector is still a problem in the droplet detection process. Common approaches to collecting droplets include polyester card [29], Carmelite paper [30], silicone oil approach [31] and water sensitive paper. Due to its high sensitivity to water and simple layout, water sensitive paper is most widely used in droplets detection. As can be always seen that, before using water sensitive paper for inspection, we need to place the water sensitive paper on the target part and fix it with a tool to prevent the wind from moving it. After the droplets deposition, it was taken back and stored in a dark, sealed and dry environment. Finally, the water paper and statistical droplet parameters were observed by a microscope or other equipment [32]. The process needed high work intensity but large human error. What’s worse was that the water-sensitive test paper was easily contaminated by oil and water and it was difficult to play a great role in an environment with high air humidity, such as paddy fields.

From the literature review, it can be found that the defects of the above-mentioned approaches are mainly reflected in (1) The droplets collection process is complicated, and the intelligent level of collection equipment is low. Meanwhile, the adaptability to illumination change is weak, and it cannot realize one-click rapid collection; (2) The high cost of materials and low utilization rate make it impossible to realize a repeated use, and its functions in an environment with high humidity are very limited; (3) The error of droplets image processing algorithm is relatively large, and it is unable to accurately and effectively detect droplets. Especially for adhesive droplets, there aren’t very effective judgment and separation methods until now.

## 3. Materials and Methods

### 3.1. Intelligent Collection Node Setup

#### 3.1.1. Design an Intelligent Collection Node Based on Vision Sensor for Droplets Deposition Image

**Figure 1:**
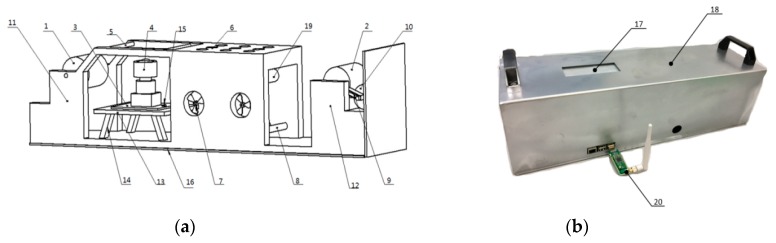
Collection device structure. (**a**) Internal structure; (**b**) External structure. Note: 1. Released reel; 2. Collection reel; 3. Raspberry pi 3b; 4. CSI camera; 5. acquisition guide shaft; 6.Ventilation hole; 7. Fan; 8. Heating tube; 9. Belt; 10. Motor; 11. Released bracket; 12. Collection bracket; 13. Storage platform; 14. Fixed tripod; 15. Illuminance sensor; 16. Shock absorbing fixed table; 17. Droplets deposition hole; 18. Environmental semi-closed shell; 19. Heating guide shaft; 20. Wireless module.

The intelligent collection node is a closed detection device, as shown in Figure 1, including a shock absorption fixed table, an environmentally friendly semi-closed shell, a droplet detection system, a drying box, an oil paper release device, an oil paper collecting device and a T- 310 achromatic oil paper. It boasts a wide use in real-time acquisition and uploading droplet image data, such as fixed bracket, CMOS Sensor Interface (CSI)camera, illumination sensor and Raspberry pi 3b, which are all set on the fixed bracket. The heating tube is installed under the drying box with its front surface and upper surface embedded a convection fan and a venting hole respectively. The drying box can quickly dry the paper attached to the droplets and make it back to its original state for reuse. We mount the heating guide shaft inside the drying box in an attempt to facilitate the transmission of the achromatic oil paper. We install the release reel and the collection reel respectively inside the release device as well as the collection device of the oil paper, and the collection reel is connected to a motor, which drives the achromatic oil paper to move from the release device to the collection device.

#### 3.1.2. Software Control Process

**Figure 2:**
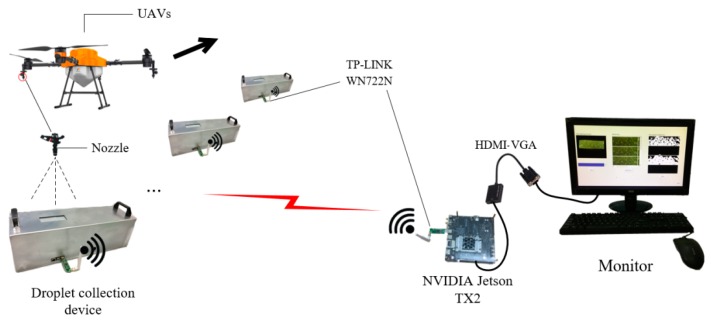
Scene structure of system work. UAV: Unmanned Aerial Vehicle

1. Reasonable software control algorithm is necessary for system automation. The control algorithm of the node consists of 4 parts:When droplet fall on its deposition hole, the T-310 achromatic oil paper reacts with the droplet and becomes transparent, thereby increasing the amount of light transmission in the semi-closed device environment and allowing the light sensor to detect the increase in the light value. Then, the system waits for 10 s to maximize the reaction. Next, turn on the CSI camera to acquire the droplet deposition image and upload it which along with illuminance data to the human-computer interaction system in the NVIDIA Jetson TX2 (NVIDIA Corporation, Santa Clara, California, USA) through the WIFI-WN722N module, as shown in Figure 2.
2. After collecting the droplet image, we turn on the motor to drive the achromatic oil paper to move to a specified distance, so that they can place the achromatic paper with droplet in the drying box.
3. Turn off the motor and turn on the relay of the drying box at the same time so as to keep the fan and the heating tube in working conditions. The drying process will last for the 30 s to restore the dry achromatic oil paper to its original color.
4. Turn on the motor to move the achromatic paper to a specified distance, and the roll paper collection device gathers the restored achromatic paper for reuse.

### 3.2. The Mathematical Model of T-310 Achromatic Ink Thickness and Light Intensity

#### 3.2.1. T-310 Achromatic Oil Paper Droplets Deposition Carrier

T-310 achromatic ink has stable properties and is not susceptible to deterioration. The ink layer is covered with a large number of micron-sized micropores. When it’s dry, it is white and opaque, and its light transmittance will be much better after contact with water. During evaporation, the ink returns to white. At this time, the light transmission achieves deterioration, which results in light reflection and scattering. Therefore, it’s of great practical value to design a highly efficient, stable and low-cost droplet deposition image carrier by combining T-310 the characteristics of the achromatic ink.

It is the silicone paper that has high tensile strength, transparency, and thin thickness, so it’s very suitable as T-310 achromatic ink printing substrate. Generally, the silk screen printing process of T-310 achromatic ink: 1. we take some T-310 achromatic ink, and then dilute it with a small amount of distilled water; 2. we can print the diluted ink on the silicone oil paper with a 70 T/cm, 0.001 cm thickness screen (water-based mesh). When the silicone oil paper is covered, the ink becomes a white film after drying; 3. If we need to increase the hiding power, we could add more layers.

According to Lambert-Beer law, under the same light intensity $I_{o}$, the absorbance A (Lx) of the uniform medium to light depends on the thickness L (cm) of the medium, which can very ideally absorb photons in the optical path. Obviously, the greater the thickness of the light passing through the object, the more light is absorbed, which will lead to a smaller transmitted light $I_{t}$. Then the following relationship will be established:

(1) $$A=\log_{10}\frac{I_{o}}{I_{t}}=KcL,$$

where K is the absorption coefficient of the medium and c is the concentration. Commonly, *c* is a constant when T-310 achromatic ink is dry. Therefore, we can set a constant τ as Kc. At this time, the Equation (1) can be transformed into:

(2) $$L=\frac{1}{\tau }\log_{10}\frac{I_{o}}{I_{t}}.$$

This indicates that the thickness of the T-310 achromatic ink becomes larger and can absorb more light as light enters the T-310 achromatic ink. The result shows that the light intensity in the detection box reduces, making the contrast between the acquired spot and the surrounding color larger. Hence, we can get a better segmentation effect. However, if the thickness is too large, the ink would be choppy after drying, thereby forming an interference crack.

#### 3.2.2. The Generation of Droplet Spot

**Figure 3:**
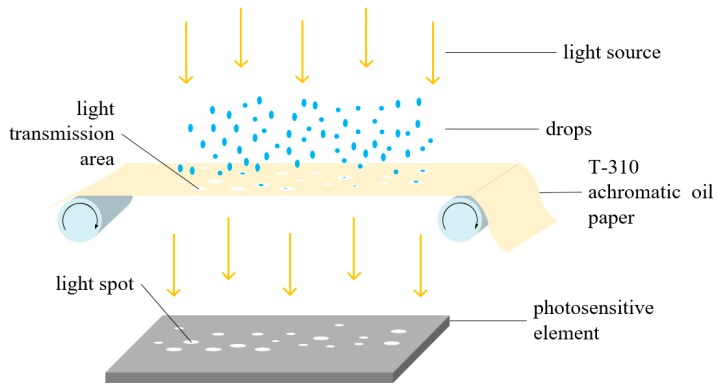
The principle of the generation of droplet spot.

When some of the droplets fall on the T-310 achromatic oil paper carrier, it will continue to diffuse to the periphery due to diffusion and penetration, and react with the T-310 achromatic ink to form a light-transmitting region at the falling position, which can be characterized by a droplet. When external light enters the detection device of the droplets, a spot can be formed on the photosensitive element of the CSI camera due to the linear propagation of the light. We utilize CSI camera as a way to obtain a spot image on the carrier, and thus they can obtain the droplets deposition effect parameter through the analysis of the spot image. The scene description is shown in Figure 3.

In test of the droplet deposition, the device is placed at a position where the droplet drops after the UAVs passes. The next step is to put the T-310 achromatic oil paper on the release reel, pull out parts and make it pass through the acquisition guide shaft as well as to collect the reel from the above CSI camera and the bottom of the heating guide shaft. When the T-310 achromatic oil paper passes through the top of CSI camera, we place it close to the droplets deposition hole for better contact with the droplets.

#### 3.2.3. Light Adaptation Based on Reinforcement Learning

In order to extract the subsequent droplet feature information favorably and eliminate noise errors, we convert the original image data from RGB space to YCbCr space, and use high-pass filter to denoise the droplet image.

Equation (2) obtains the optimal ink thickness within unchanged ambient illumination, with the best droplet image contrast and segmentation. However, in reality, the ambient illumination is always changing slightly, which makes it impossible to obtain the best segmentation by continuously changing the ink paper of different thicknesses. Therefore, we establish an illumination adaptive model with Sarsa algorithm to reduce the impact of illumination changing on the precise picking of droplets.

The online illumination adaptive processing method generally comprises two parts: (1) collecting related parameters; (2) adaptively identifying the target within different illumination with the collected parameters. The first part firstly obtains the droplet image through the camera. Then manually adjusting the image contrast, thus obtaining the optimal segmentation within certain illumination. The segmentation may be objectively evaluated with feature fusion [33], which calculate the average spacing of mismatched pixel points as the standard spacing of the best segmentation. Then the contrast within real-time illumination will be obtained, and the optimal contrast adjustment parameter, which is the degree of quantization of the contrast adjustment [34] and defined as c in this paper, will also be obtained with Reinforcement Learning algorithm. The second part is about the application. Adaptive adjustments will be made to the droplet image contrast within different illumination with the parameters obtained in the first part. It must be noted the contrast adaptive adjustment with Sarsa learning occurs on the NVIDIA Jetson TX2 which isn’t a real-time system, so the learning time is not closely related to droplet parameters acquisition. The Sarsa learning is ans′a′on-policy temporal difference algorithm in Reinforcement Learning, which conducts iterative update with a quintuple (*s*, *a*, *r*, *s′*, *a′*). Specifically, s indicates the current state; *a* indicates the action selected in the current state; *r* indicates the reward for action *a*; *s′* and *a′* indicate the subsequent states and actions, respectively. The behavior value function in the Sarsa algorithm is iterated by the actual Q value, and the $Q^{*}\left(s,a\right)$ is obtained by the iterative rule within the Equation (3):

(3) $$Q\left(s_{t},a_{t}\right)\leftarrow Q\left(s_{t},a_{t}\right)+\alpha \left[r_{t}+\gamma Q\left(s_{t+1},a_{t+1}\right)-Q\left(s_{t},a_{t}\right)\right].$$

**Figure 4:**
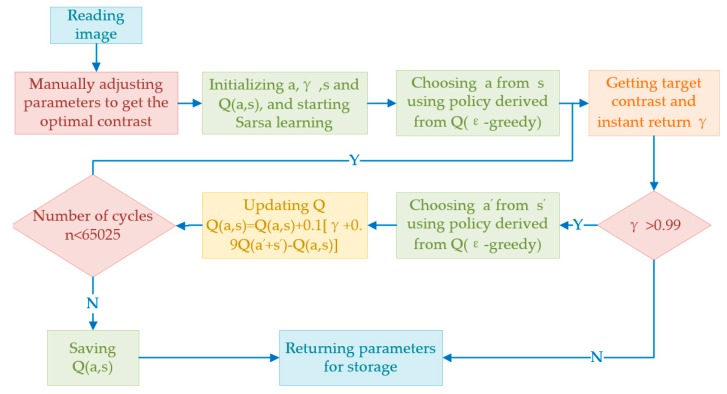
The Sarsa learning flow chart.

In the equation, $Q\left(s_{t},a_{t}\right)$ indicates the Q value at time t, with the initial value as 0; $a_{t}$ indicates the current action; $r_{t}$ indicates the value of the reward function (the ratio of the real-time average spacing to the standard spacing in this paper); γ indicates the discount factor (the importance of future reward, as compared with the current reward); α indicates the learning rate (0≤α≤1, 0<γ≤1). This paper sets α=0.1 and γ=0.9 in order to balance the convergence speed and stability. This paper takes ε−greedy as the exploration strategy, taking advantage of the balancing knowledge exploration of the unknown and making full use of existing empirical knowledge of ε-greedy. In this case, Figure 4 shows the complete process of the Sarsa learning.

### 3.3. The Modified Morphology Algorithm of Droplet Measurement

#### 3.3.1. Separation of Adhesive Droplets

The shape degree E of the target boundary [35] was introduced as the basis for the determination of the adhesive droplets:

(4) $$E=\frac{4\pi A}{L^{2}},0<E\leq 1,$$

where $A=\left\{A_{1},A_{2},\ldots ,A_{n}\right\}$ is the pixel value of the target droplet connection region, and $L=\left\{L_{1},L_{2},\ldots ,L_{n}\right\}$ is the peripheral pixel value of the droplet connection region. When the target droplet is a circle, then E = 1. Usually, the shape degree threshold e is 0.5, which can also be determined by judging the difference of mean value between the shape degree of the individual droplets and the adhesive droplets [36]. In this paper, we use the latter approach to select 1000 individual droplets and adhesive droplets on the achromatic oil paper. As a result, it is statistically found that the average shape degree value of the individual droplets is 0.86, and the adhesive droplet is 0.48, where e is equal to 0.67. If an adhesive droplet has a good circularity, a large relative area and an inconspicuous dividing point, we cannot extract the shape degree accurately. Therefore, the area threshold is introduced as an aid for its feature extraction and also as a basis for determining droplets’ adhesion. The calculation formula of the area threshold d is as shown in the Equation (5):

(5) $$d=\frac{\sum_{i=1}^{n}A_{i}}{n},$$

where $A_{i}$ is area pixel value of *i*-th droplets area after binarization of the original image, and n is the number of droplets areas. Therefore, only when a certain area satisfies: $E_{i}>e$ & $A_{i}<d$, it is a single droplet, otherwise it is an adhesive droplet.

We define the adhesive droplet image as f(x, y), the structural element as b(x, y), and then define its gradient image g(x, y) as Equation (6):

(6) g(x,y)=(f(x,y)⊕b(x,y))−(f(x,y)⊖b(x,y)),

where ⊕ and ⊖ represent the grayscale morphological expansion and corrosion operations, respectively.

The operation of morphological open reconstruction is based on the geodesic expansion, and can be defined as Equation (7):

(7) $$O_{b}^{\left(rec\right)}\left(g,r\right)=D_{b}^{\left(rec\right)}\left[\left(g\circ b\right),r\right],$$

where ∘ is a morphological opening operation, r is a reference image, rec=(1,2,3,…), and $D_{b}^{\left(rec\right)}$ is the result when the morphological geostrophic expansion converges.

Similarly, the morphological closed reconstruction operation is based on geodesic corrosion and defined as Equation (8):

(8) $$C_{b}^{\left(rec\right)}\left(g,r\right)=E_{b}^{\left(rec\right)}\left[\left(g\circ b\right),r\right],$$

where $E_{b}^{\left(rec\right)}$ is the result when the morphological geodesic corrosion converges.

The morphological hybrid opening and closing reconstruction operation is defined as secondary reconstructions of the first opening and closing. Firstly, the utilization of the morphological open reconstruction is used to eliminate large-scale noise and irregular interference of scales smaller than the structural elements in the gradient image, thereby facilitating the operation of the morphological closed reconstruction to remove dark noise and irregular interference smaller than the structural elements. The formula is as follows:

(9) $$g_{b}^{\left(rec\right)}=C_{b}^{\left(rec\right)}\left[O_{b}^{\left(rec\right)}\left(g,r\right),r\right].$$

#### 3.3.2. Droplet Deposition Parameters

We count the number of droplets N on the condition that the adhesive portion is broken and the small protruding portion is removed. The droplet coverage density *K* is an important parameter that can judge the quality of the spray, and its definition formula is as follows:

(10) $$K=\frac{N}{S}\;,$$

where *S* is the total area of the droplets image, cm^2^. With regard to the calculation of the droplet coverage, it could be expressed on the droplet binary image with the help of the area of the droplets in the image and the percentage of the area of the entire image. The formula for calculating the droplets coverage rate C is as follows:

(11) $$C=\frac{S_{\left(PIX=1\right)}}{S_{\left(PIX=0,\;1\right)}}\times 100\%,$$

where $S_{\left(PIX=1\right)}$ represents the area of the binary image with a pixel value of 1. Similarly, $S_{\left(PIX=0,\;1\right)}$ represents an area in which the pixel value is 0 or 1.

#### 3.3.3. Modified Marker Controllable Watershed Segmentation Algorithm

The droplets segmentation algorithm in this paper is shown in Table 1.

**Figure 5:**
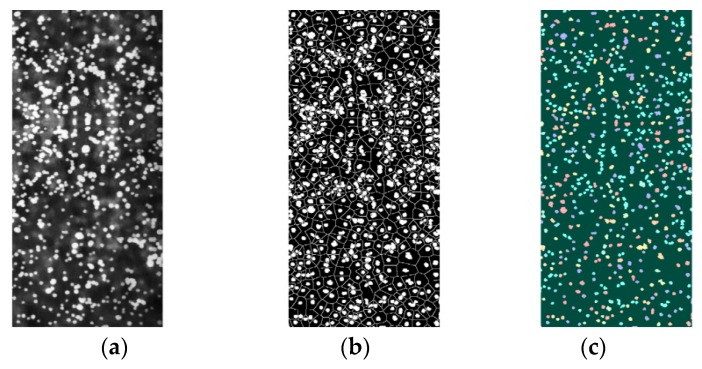
The effect of image segmentation. (**a**) Original gray image; (**b**) Markers and object boundaries image; (**c**) LRGB image.

In the segmentation function, dark areas are the image of objects we are attempting to segment. The computed foreground markers are connected blobs of pixels within each of the objects, and the background markers are also pixels that don’t belong to any object. Modify the segmentation function so that it only has a minimum at the foreground and background marker locations. The result is shown in Figure 5.

In Figure 5, the MMCWS algorithm is capable of separating the adhesive droplets in the image and marking each droplets area. This approach is suitable for the segmentation of large flow droplets, but some adhesive droplets are still excessively segmented or undivided. Part of the reason is that the angle between the droplets and the carrier is small, producing a slender tail, which results in the shape degree is too small to be excessively divided. Another reason is those small droplets have a tiny effect on the shape degree of the area when they are connected to large droplets.

## 4. Experiments and Results

### 4.1. Verification of the Relationship between the Thickness of Achromatic Oil Paper and the Light Intensity

**Figure 6:**
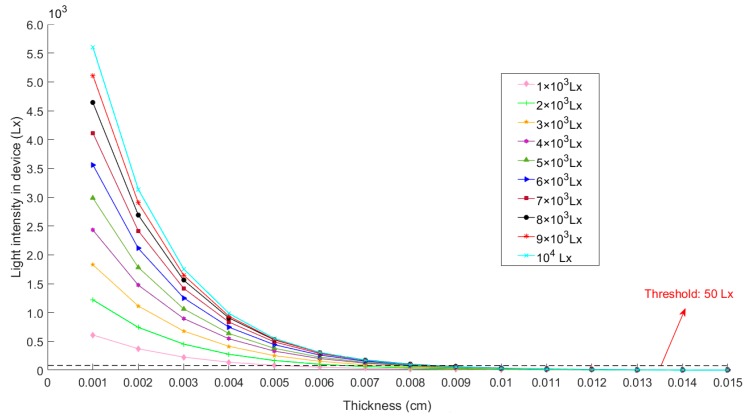
The variation of the light intensity value in the test box with the thickness of the ink under different light intensities.

To testify the relationship between the intensity of light and the thickness of ink at a specific light intensity, we set the ink thickness to 15 categories, namely, 1 layer (0.001 cm), 2 layers (0.002 cm), 3 layers (0.003 cm) ... 14 layers (0.014 cm), 15 layers (0.015 cm), and simulate the change of ambient light through LED backlighting scheme with adjustable brightness, respectively testing the light intensity value under the condition of 1 × 10^3^ Lx, 2 × 10^3^ Lx, 3 × 10^3^ Lx … 9 × 10^3^ Lx, 1 × 10^4^ Lx light intensity in the box, and the result is shown in Figure 6.

Figure 6 shows that under the same light intensity, the greater the thickness of the ink, the smaller the light intensity value in the detection box. When the thickness exceeds 0.011 cm, the light intensity approaches zero and remains unchanged, which is approximately in accordance with the Lambert-Beer law. When the ink has the same thickness, the stronger the light source, the larger the light intensity value in the detection box. It has been confirmed that when the light threshold in the detection box is lower than 50 Lx, the droplet separation effect will keep unchanged by $I_{o}$. Therefore, we set the transmitted light $I_{t}=$ 50 Lx, the mean$\;\tau_{m}$ of τ, which is defined under each light condition as shown below.

(12) $$\tau_{m}=\frac{1}{n}\sum_{i=1}^{n}\frac{1}{L_{i}}\log_{10}\frac{I_{i}}{50},$$

where $I_{i}$ is the light intensity of the *i*-th test. $L_{i}$ is the ink thickness under the condition that the transmitted light intensity is 50 Lx in the *i*-th test. Through reading each parameter into Equation (12) by Figure 5, we get $\tau_{m}$ = 257.11, so the relationship between the light intensity $I_{o}$ and the ink thickness threshold L can be expressed as Equation (13):

(13) $$L=\frac{1}{257.11}\log_{10}\frac{I_{o}}{50}.$$

**Figure 7:**
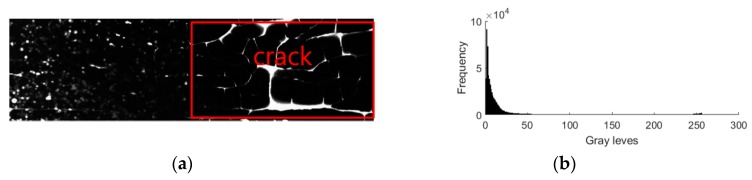
(**a**) Droplets image of 1.5 × 10^−2^cm ink; (**b**) its grayscale pixel distribution curve.

In the process of collecting the droplets images, the appropriate ink thickness should be selected in order to highlight the characteristics of the droplets spot based on the ambient lighting conditions, which is the key to improving the accuracy of the droplets detection in this system. For example, when the thickness of the ink is set to 1.5 × 10^−2^ cm, the ink may interfere with the crack to affect the efficiency of the droplet detection and the result is shown in Figure 7 under the condition of 3 × 10^3^ Lx.

### 4.2. Illumination Adaptive Simulation Analysis Based on Reinforcement Learning

We change the illumination intensity with adjustable HF-HX8036 led light. As this paper aims to verify the feasibility of the adaptive algorithm, therefore this paper only tests the adaptive parameters of 100 illumination values within the ambient illumination of $\left[3.50\times 10^{3}\mathrm{Lx},3.60\times 10^{3}\mathrm{Lx}\right]$ and collects the droplets from selected ink paper with relevant thickness based on the Equation (13). Reinforcement Learning can be performed within the currently changed illumination, which enables the program to automatically explore the optimal strategy for the optimal contrast value that minimizes the average spacing of unmatched pixels.

**Figure 8:**
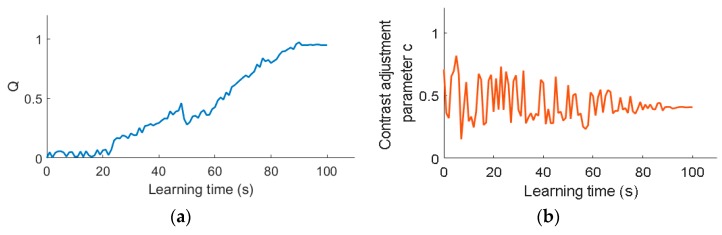
Reinforcement Learning process of related parameters. (**a**) The curve of Q value; (**b**) The curve of contrast adjustment parameter c.

Figure 8 shows the learning curve of the value Q curve and the contrast adjustment parameter c acquired within the illumination of $3.55\times 10^{3}\;\mathrm{Lx}$.

Figure 8a shows that the Q value is initialized to zero at the beginning of learning. Although the Q value fluctuates continuously with the learning time, the value Q generally keeps going up towards 1, thus avoiding the defect that the Q value takes a locally optimal solution in a single direction, which indicates that the Sarsa algorithm can achieve a global optimal solution. The Figure 8b shows that the learning curve of the number between 0 and 1 randomly selected at the beginning fluctuates greatly during the learning process, for the Sarsa algorithm is continuously exploring in the early stage of learning. Nonetheless, the optimal solution is being gradually obtained, for the later exploration gradually becomes utilization.

**Figure 9:**
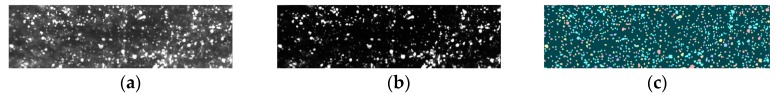

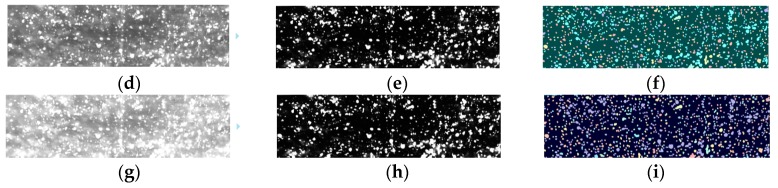
The results of adaptive under different illumination intensity. (**a**–**c**) are respectively original droplets image, optimum contrast image and segmented result within the illumination of $3.50\times 10^{3}\;\mathrm{Lx}$; (**d**–**f**) are respectively original droplets image, optimum contrast image and segmented result within the illumination of $3.55\times 10^{3}\;\mathrm{Lx}$; (**g**–**i**) are respectively original droplets image, optimum contrast image and segmented result within the illumination of $3.60\times 10^{3}\;\mathrm{Lx}$.

The next step is to apply the values to achieve adaptive recognition in different illumination. The system automatically adjusts the optimal contrast value in real time based on the collected illumination information in the adaptive recognition process, thus obtaining the best segmentation. In order to verify the effectiveness of the adaptive algorithm, we apply it to process the same oil paper with droplets within different illumination that are $3.50\times 10^{3}\;\mathrm{Lx}$, $3.55\times 10^{3}\;\mathrm{Lx}$ and $3.60\times 10^{3}\;\mathrm{Lx}$, respectively. The droplets image with the best contrast and their segmented result as shown in Figure 9.

Figure 9 shows that our algorithm can effectively adapt to changes in illumination and adjust the contrast adjustment parameters c to obtain the best segmentation result when illumination increases from $3.50\times 10^{3}\;\mathrm{Lx}$ to $3.60\times 10^{3}\;\mathrm{Lx}$. The contrast adjustment parameters c of (b), (e), and (h) are 0.31, 0.40, and 0.53, respectively. The coverage density K of (c), (f), and (i) are 147 n·cm^−2^, 152 n·cm^−2^, and 151 n·cm^−2^, respectively; The False Positives of them are serially 1.87%, 1.69%, and 1.92%.

### 4.3. Comparative Test and Analysis of Distribution Characteristics for Droplet Deposition

**Figure 10:**
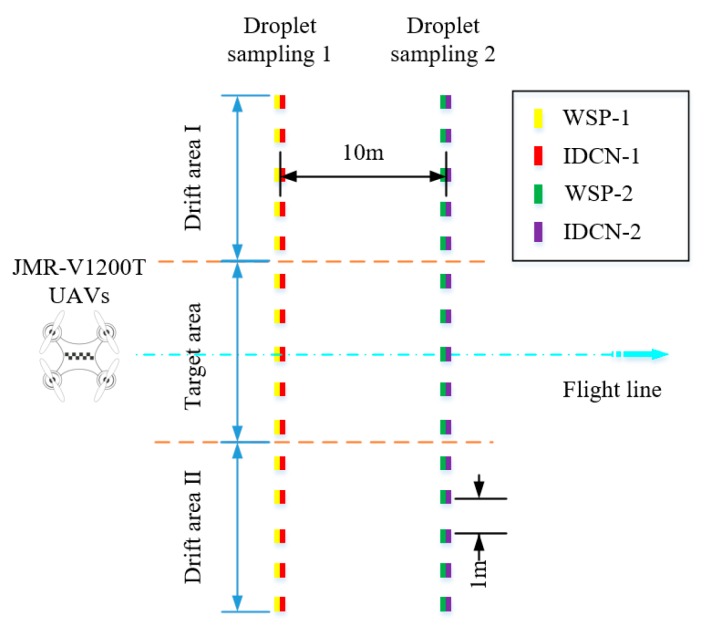
Schematic diagram of the test. WSP: Water Sensitive Paper; IDCN: Intelligent Droplet Collection Node.

In order to qualitatively analyze the influence of different droplet collection schemes on the distribution characteristics of droplet deposition, we design the experimental scheme shown in Figure 10. In the natural working environment, we research the distribution of droplets deposition in the sampling of Water Sensitive Paper (WSP) and Intelligent Droplet Collection Node (IDCN) at different working heights and speeds. The test location is Huashan Sports Ground of South China Agricultural University (113°34′ E, 23°15′ N), which is an open area and has no wind disturbance in the environment.

We select the JMR-V1200T six-rotor plant protection drone, of which the technical parameters are shown in Table 2.

There are two droplets sampling belts in the working area with an interval of 10 m between them. Each sampling belt is provided with a target area of UAVs and two drift areas on either side of the target area. According to the product parameters of UAVs, the width of the target area and the drift area is set to 5 m with the space of the sampling points on each sampling belt being 1 m, and there are 32 sampling points in sampling belts in total. At the same time, a T-310 achromatic oil paper droplets detection device and a water sensitive test strip sample are placed at each sampling point.

Before the test, we measured the ambient light intensity to be 3.54 × 10^3^ Lx. According to Section 4.1 and Equation (13), the light intensity in the test box will be less than 50 Lx only when L ≥ 0.0072 cm is satisfied. Therefore, the ink thickness is set to 0.01 cm in this test.

We use the ATK-S1216 GPS system (Guangzhou Xingyi Electronic Technology Co., Ltd., Guangzhou, Guangdong, China)to receive the latitude and longitude of each sampling point as well as the flight Line of the drone, which are converted into geodetic coordinates by the seven-parameter method [37,38].

After completing the drone spraying test, we should measure the operating parameters of each test drive, as shown in Table 3.

**Figure 11:**
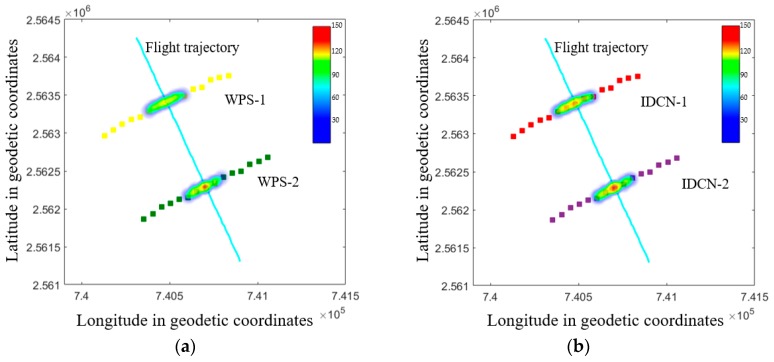

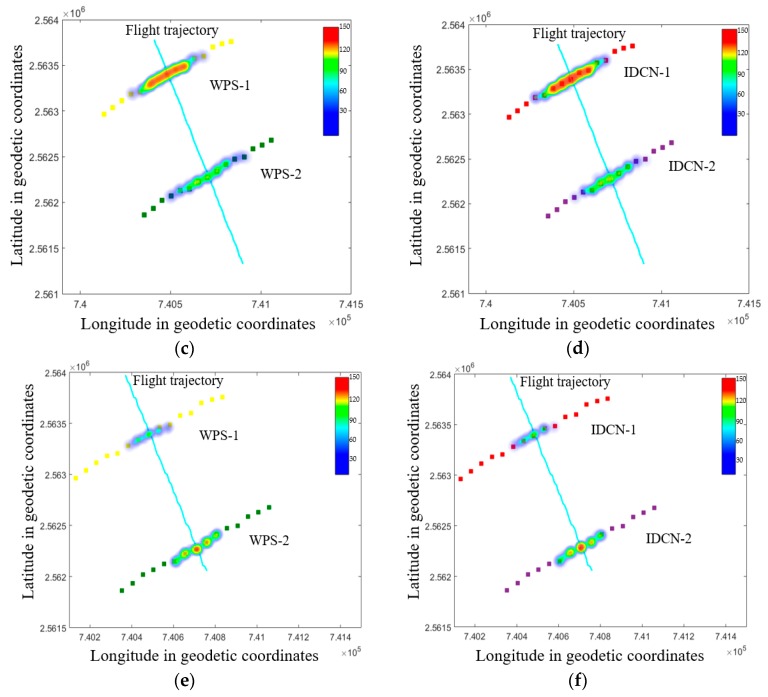
Heat map of droplet coverage density in each test. (**a**) Test1-WSP. (**b**) Test1- IDCN. (**c**) Test2-WSP. (**d**)Test2- IDCN. (**e**) Test3-WSP. (**f**) Test3- IDCN.

The WSP of each sampling point is collected and analyzed by the image processing software Deposit Scan so as to obtain the droplets deposition parameter. At the same time, we also take advantage of the PC software designed by us to detect the droplets images of T-310 achromatic oil paper at each sampling point in real time and obtain the deposition parameter associated with them. Finally, the droplets coverage density value is selected and visualized in the form of a heat map, as shown in Figure 11.

When the flight speed of Figure 11a–d are basically similar, the flying height of test 2 is 5.05 m; the heat map shows that the droplet have covered the target area, and the droplets are also dropped in the drift area; while test 1 is 1.41 meters and only droplets could be detected in the target area. The results show that as the height increases, the vertical wind field above the sampling point is weakened, and the amount of droplet deposition in the target area would gradually decrease. Figure 11a,b,e,f depict that the flight speed does not have a significant effect on the amount of droplets deposition in the target area at the same flight height. Finally, we model and make the analysis of the coverage density values of IDCN and WSP and find that the fitting accuracy of both is 0.91.

## 5. Conclusions

In the research, we design an intelligent vision sensor node that can adapt to the changes of light intensity in the environment, enabling rapid collection of large-scale droplets deposition parameter. Specifically, a morphology based segmentation algorithm for large flow droplets is designed. We first employ the shape degree and area threshold as the basis for the determination of droplets’ adhesion. Next, we make use of the marker-controlled watershed segmentation to separate the adhesive droplets in the image, and each droplets area is successfully segmented and marked. Then, we accurately estimate the quantity of droplets, with its unit coverage density in the node’s test window.

In final experiment, the droplet coverage density of WSP and IDCN approaches is shown in the heat map, and the fitting accuracy of both is 0.91. Such results demonstrate that our approach is feasible and makes up for the shortcomings of the traditional measurement approach, such as poor continuity of sample data, cumbersome processing approach and non-recyclable materials. It improves the accuracy of the detection of droplets deposition characteristics and enables the rapid measurement of the droplets deposition law of UAVs application spray.

Possible work in the future is as follows: (1) The intelligent collection node can be optimized by providing a fuzzy control algorithm for the adaptive calibration of internal light source; (2) Further research of droplet segmentation approaches based on deep learning to further improve the accuracy of segmentation; (3) Combination of the use of other spray system, such as ground spray system with different nozzles. We also believe that it will be interesting to extend the scope of the study to the measurement and statistical analysis of droplet patterns.

## Figures and Tables

**Table 1:** Modified marker controllable watershed segmentation algorithm.

**Input**: droplets’ YCbCr image	
**Output:** marked droplets image $F_{m}$, droplets’ number N, Coverage C, Coverage density K	
1	Read the Image and Convert it to binary image with Otsu method F←readimage() $F_{g}\leftarrow \mathrm{Otsu}\left(F\right)$
2	Use the Gradient Magnitude as the Segmentation Function $h_{y}\leftarrow \mathrm{sobel},h_{x}\leftarrow h_{y}{}^{\sim }$ $\left(I_{x}{}^{2},I_{y}{}^{2}\right)\leftarrow \mathrm{filter}\left(F_{g},h_{x},h_{y}\right)$ $F_{1}\leftarrow \mathrm{sqrt}\left(I_{x}{}^{2}+I_{y}{}^{2}\right)$
3	Judging sticky droplets. $E,e\leftarrow \frac{4\pi A}{L^{2}}$,$d\leftarrow \frac{\sum_{i=1}^{n}A_{i}}{n}$ for i←1 to n do If $E_{i}>e$ and $A_{i}<d$ go to step 4 else go to step 5 end
4	Mark Foreground Objects, and count the number of droplets $F_{2}\leftarrow \mathrm{reconstruct}(\mathrm{dilate}(\mathrm{reconstruct}\left(\mathrm{erode}\left(F_{1}\right)\right)$)) $outputN\leftarrow count\left(F_{2}\right)$ $F_{3}\leftarrow Seedfill\left(F_{2}\right)$
5	Compute Background Markers, and calculate coverage $F_{4}\leftarrow \mathrm{binarize}\left(F_{3}\right)$ $\mathrm{output}\;C\leftarrow \frac{S_{\left(PIX=1\right)}}{S_{\left(PIX=0,1\right)}}\left(F_{3}\right)\times 100\%$ $\mathrm{output}\;K\leftarrow \frac{N}{S}$
6	Compute the Watershed Transform of the Segmentation Function $F_{5}\leftarrow \mathrm{Imposemin}\left(F_{1},\mathrm{watershed}\left(F_{4}\right)\right)$ (modify the gradient magnitude image) $F_{6}\leftarrow \mathrm{watershed}(F_{5})$
7	$F_{7}\leftarrow \mathrm{labeltorgb}(F_{6})$

**Table 2:** Product parameter of JMR-V1200T.

Product Parameter	Norms and Numerical
Size/cm × cm × cm	110 × 125 × 68
load/kg	10
Flight speed/(m·s^−1^)	3–6
Spraying width/m	3–8
Flight height/m	1.2–6
Flight time/min	10–14

**Table 3:** Real-time operation parameters of UVAs on each test.

Test	Approach	Flight Speed (m·s^−1^)	Flight Height (m)	Average Coverage Density (n·cm^−2^)	
				Sampling Belt 1	Sampling Belt 2
1	WPS	3.11	1.41	73	99
	IDCN			79	93
2	WPS	3.09	5.05	137	65
	IDCN			128	68
3	WPS	5.89	1.46	59	89
	IDCN			63	85
